# Vehicle Windshield Wiper Fluid as Potential Source of Sporadic Legionnaires’ Disease in Commercial Truck Drivers

**DOI:** 10.3201/eid2804.210814

**Published:** 2022-04

**Authors:** Julieta Politi, Andreu Queralt, Natalia Valero, Maria T. Martín-Gómez, Raquel González Durán, Elena Parra, Sara Sabaté Camps, Ingrid Avellanés, Anna Hernández-Pineda, Eva Masdeu, Cristina Rius, Dolores Álamo-Junquera

**Affiliations:** Parc de Salut Mar, Barcelona, Spain (J. Politi);; Public Health Agency of Barcelona, Barcelona (J. Politi, A. Queralt, N. Valero, R. González Durán, E. Parra, S. Sabaté Camps, I. Avellanés, A. Hernández-Pineda, E. Masdeu, C. Rius, D. Álamo-Junquera);; Hospital Universitari Vall d’Hebron, Barcelona (M.T. Martín-Gómez);; Centro de Investigación Biomédica en Red de Epidemiología y Salud Pública, Barcelona (C. Rius);; Universitat Autònoma de Barcelona, Barcelona (C. Rius);; Institut d’Investigació Biomèdica Sant Pau (IIB Sant Pau), Barcelona (C. Rius);; Universitat de Barcelona, Barcelona (D. Álamo-Junquera)

**Keywords:** Legionnaires’ disease, Legionella, bacteria, vehicle, windshield wiper fluid, surveillance, environmental exposure, occupational health, commercial truck drivers, Barcelona, Spain

## Abstract

Sporadic Legionnaires’ disease is frequently detected in commercial truck drivers. We report 2 sporadic cases of this disease in Barcelona, Spain, that occurred during December 2019 and September 2020. Laboratory findings were consistent with windshield wiper fluid without added screen wash as a possible source of infection for both cases.

Legionnaires’ disease is a severe form of acute pneumonia caused by inhalation of aerosols containing *Legionella* bacteria. Most *Legionella* infections are related to contaminated artificial water systems. Systems with warm water (35°C), stagnation, and lack of disinfection and maintenance can lead to proliferation of *Legionella* spp. (*1*). Cooling towers, warm water systems, and whirlpool spas are well-established sources of infection (*2*). However, in most sporadic cases, the source of infection remains unknown (*3*).

Commercial truck drivers are at increased risk for Legionnaires’ disease (*4*–*7*). Exposures related to the vehicle are usually considered secondary to outside sources in industrial areas, such as cooling towers, and are seldomly investigated, despite some studies suggesting them as potential sources (*4*). Using windshield wiper fluid without added screen wash has been identified as a risk factor for Legionnaires’ disease in commercial drivers in a previous case‒control study (*4*). In addition, *Legionella* spp. can grow in windshield wiper fluid that does not contain screen wash. However, no studies have epidemiologically confirmed the fluid as the source of infection (*8*). We report 2 cases of Legionnaires’ disease cases diagnosed by urine antigen testing (UAT) linked to detection of the bacteria in the windshield wiper fluid.

## The Study

In December 2019, the Public Health Agency of Barcelona (PHAB) received a case report of Legionnaires’ disease in a 59-year-old man. Onset of symptoms had begun a week before diagnosis. On December 13, the patient sought care at a hospital, and a diagnosis of Legionnaires’ disease was made by UAT. A respiratory sample was not available because of the lack of productive secretions. The patient was hospitalized briefly, and his clinical course was unremarkable. After discharge, he completed the remaining course of antimicrobial drug therapy and proceeded favorably to cure.

After the case was reported, public health nurses contacted the patient to complete a structured epidemiologic questionnaire that included demographic data, personal risk factors, activities, and potential exposures during the 14 days before illness onset. The patient smoked and had a medical history of hypertension and type 2 diabetes. He worked as a commercial truck driver, and his driving route included merchandise pickup at an industrial area once a day. The truck used for work was self-owned; the patient had purchased it secondhand ≈1.5 years before illness. He reported that the vehicle had been unused for several months before the purchase and denied using screen wash in the windshield wiper fluid.

No other cases of Legionnaires’ disease reported during the same period were related to his residence or driving areas. Two weeks after the interview, environmental inspectors from PHAB sampled the fluid that remained in the windshield wiper tank of the truck. The 2,000-mL sample of water was collected and stored in a sterile container (Deltalab, https://www.deltalab.es) treated with sodium thiosulfate. The PHAB laboratory analyzed the sample for *L. pneumophila*. Culture results using *Legionella* selective media, according to the ISO 11731:2017 protocol (https://www.iso.org), were negative. However, the sample was also analyzed by using real-time PCR (Appendix). Testing showed that the fluid was PCR positive for *L. pneumophila* (Table). Laboratory test results and cleaning procedures to follow for the windshield wiper tank were explained to the driver. Public health inspectors also recommended adding screen wash fluid to the windshield wiper fluid regularly.

**Table:** Results of *Legionella* testing in clinical and environmental specimens related to commercial truck drivers, Barcelona, Spain, 2019–2020

Case-patient	Specimen	Test	Test results	Interpretation
1	Urine	Urinary antigen	Positive	*Legionella pneumophila* serogroup 1
	Windshield wiper fluid	PCR	Positive	*L. pneumophila*
	Windshield wiper fluid	Culture	Negative	NA
2	Urine	Urinary antigen	Positive	*L. pneumophila* serogroup 1
	Windshield wiper fluid	PCR	Negative	NA
	Windshield wiper fluid	Culture	Positive	*Legionella* spp.

*NA, not applicable.

On September 17, 2020, a new case of Legionnaires’ disease was detected by UAT in commercial truck driver. A respiratory sample was unavailable because of lack of respiratory secretions. The patient was a 58-year-old man who smoked and had a medical history of recently diagnosed chronic obstructive pulmonary disease. He was hospitalized briefly and was successfully treated with antimicrobial drug therapy. His driving routes included industrial areas frequently located within Barcelona. No further cases that could be epidemiologically linked to this case were identified during the same period.

The patient used the truck daily and denied adding screen wash to the windshield wiper fluid; he also reported that the windshield wiper fluid had not been changed for >6 months. On the basis of previous case experience, we obtained a sample of the windshield wiper fluid. Analysis of the sample was performed in the PHAB laboratory according to the stated standards. Although we were unable to detect *L. pneumophila* by culture or real-time PCR, *Legionella* spp. was detected by culture at a concentration of 6.3 × 10^3^ CFU/L (Table). Consequently, public health inspectors recommended cleaning procedures to disinfect the windshield wiper tank, together with adding screen wash fluid to the windshield wiper fluid regularly.

## Conclusions

We report 2 sporadic cases of Legionnaires’ disease in commercial truck drivers in which the laboratory findings were consistent with, but not exclusive to, the windshield wiper fluid as the source of infection. The etiologic agent was presumed to be *L. pneumophila* serogroup 1 (*Lp*1) because both patients were given a diagnosis by UAT, which detects only *Lp*1. DNA of *L. pneumophila* (unknown serogroup) was found in the windshield wiper fluid for the first case, and *Legionella* spp. was cultured from the fluid for the second case. Although the presence of *Lp*1 was not confirmed in the windshield wiper fluid for either case, laboratory findings indicated that the flid was a potential source for both cases.

Previous studies have identified windshield wiper fluid without screen wash as a potential risk factor for Legionnaires’ disease (*4*). Furthermore, 2 previous studies have identified *Legionella* spp. in windshield wiper fluid (*8*,*9*), confirming that the bacteria can survive in this medium and the fluid as a possible source of infection. These results are consistent with our observations for the windshield wiper fluid for the second case described. Despite these observations, transmission from this source has not been epidemiologically confirmed. Our findings strengthen the epidemiologic connection between windshield wiper fluid as a source of infection for truck drivers.

Several vehicle-related sources have been described as confirmed or potential sources of Legionnaires’ disease, although these sources were infrequently considered and difficult to investigate (*3*,*10*,*11*). Kanatani et al. identified *L. pneumophila* in road puddles (*12*) and hypothesized that bacteria reach the windshield wiper fluid tank through road splashes. Alternatively, *Legionella* spp. could reach the tank after a car wash at a contaminated installation or through a contaminated water source used to fill the tank. Warm temperatures or heat radiated from the motor of the vehicle to the water tank, along with lower or absent methanol levels in screen wash fluid, and water stagnation could favor bacterial proliferation in the windshield wiper fluid tank.

Beyond routinely investigating this source, especially in commercial truck drivers, a simple measure of adding screen wash to the fluid can be recommended. This action has the potential benefit of decreasing the risk for infection by inhibiting growth of *Legionella* spp. growth through a bactericidal effect of screen wash components, such as propanol/methanol (*9*).

In summary, our results indicate that windshield wiper fluid is a potential source of sporadic Legionnaires’ disease, especially in commercial truck drivers, and should be routinely investigated. A simple recommendation of adding screen wash to windshield wiper fluid or emptying the tank when the vehicle is unused for several months is a preventive measure likely to be effective and should be adopted by drivers.

Appendix Additional information on vehicle windshield wiper fluid as potential source of sporadic Legionnaire’s disease in commercial truck drivers.
