# Supplementary material for: Vehicle Windshield Wiper Fluid as Potential Source of Sporadic Legionnaires’ Disease in Commercial Truck Drivers
Source: Emerg Infect Dis. 2022 Apr;28(4):841–3. doi: 10.3201/eid2804.210814 (PMC8962911; doi:10.3201/eid2804.210814)
Supplement: Appendix — Additional information on vehicle windshield wiper fluid as potential source of sporadic Legionnaire’s disease in commercial truck drivers. [file 21-0814-Techapp-s1.pdf]

Article DOI: https://doi.org/10.3201/eid2804.210814

Vehicle Windshield Wiper Fluid as Potential Source of Sporadic Legionnaires' Disease in Commercial Truck Drivers

Appendix

Laboratory Methods

Culture Method

The ISO 11731 specifies culture methods for the isolation of *Legionella* and estimation of their numbers in water samples. It is the standard method for environmental laboratories in Spain. Annex J of the ISO 11731:2017 (https://www.iso.org) establishes a decision matrix to choose the methods, treatments, and culture media on the basis of the origin and characteristics of the sample. The windshield wiper fluid was classified as matrix B (water with high background).

The selected methods were direct plating and filtration with washing procedure. Direct plating was performed inoculating 0.2 mL of the sample in 3 culture plates with acid, heat, and without treatment respectively. In brief, a 1-L water sample was filtered through a 0.2-µm pore size polycarbonate membrane (Sartorius Laboratory Instruments GmbH and Co., https://www.sartorius.com) by using a peristaltic pump, and the membrane was then resuspended into 10 mL of 1:40 Ringer solution and vortexed for 3 ± 1 min to wash the microorganisms from the membrane. A 0.2-mL aliquot of the concentrate was spread on culture plates per duplicate with acid, heat and without treatment, respectively.

The culture medium was the selective Glycine-Vancomycin-Polymyxin-Cycloheximide medium (bioMérieux, https://www.biomerieux.com). Plates were incubated at 36 ± 2°C for 10 days in a humid atmosphere. Five or more presumptive colonies were subcultured onto buffered charcoal yeast extract medium (Oxoid; Thermo Fisher Scientific

Inc., https://www.thermofisher.com) and Columbia blood agar plates (Oxoid, Thermo Fisher Scientific Inc.). *Legionella* only growing on buffered charcoal yeast extract medium were further identified by using latex agglutination testing (Oxoid Thermo Fisher Scientific Inc.) according to the manufacturer's instructions. The limit of detection of the method for Matrix B samples is 25 CFU/L.

Real-Time PCR

The real-time PCR method targets the *mip* gene (macrophage infectivity potentiator) and it is based on the assay developed by Benitez and Winchell (*1*). It includes some modifications but do not imply changes in the concentration or sequence of the primers or probe. The method performed in the Public Health Agency of Barcelona Laboratory meets the criteria of Inclusiveness and Exclusivity of ISO/TS 12869:2019 and has a limit of detection of 500 genomic units/L in nonpotable water. The limit of detection was determined as the lowest number of genomic units that gave a positive result in 90% of cases.

PCR Primers and Probe

Primer and probe sets specific for *mip* gene were used as described (*1*). This gene target exists as a single copy in the *Legionella* genome (*2*).

Assay Conditions

Briefly, 200 mL of the sample were filtered in a 0.4-µm pore size polycarbonate membrane (Millipore; Merck Life Science, https://www.emdgroup.com) and then resuspended into 2.37 mL of a 30% Chelex 100 resin (Bio-Rad, https://www.bio-rad.com) and suspended in Tris-EDTA buffer. The concentrate was then lysed, extracted, and purified by using NucliSENS Lysis and Magnetic Extraction Reagents (bioMérieux). The final volume of the eluate was 150 µL and was stored at −70°C. Every series of extraction incorporates in parallel to the samples a positive extraction control ($5x10^5$ UG of *L. pneumophila* sg 1 ATCC-33152 genomic DNA into 2. 37 mL of a 30% Chelex 100 resin and suspension in Tris-EDTA buffer).

Real-time PCRs were performed by using the Roche Diagnostics LightCycler 2.0 Instrument (https://www.roche.com) under the following conditions: 40°C for 10 min, 95°C

for 10 min; followed by 45 cycles of 95°C for 10 s, 62°C for 45 s, and 72°C for 1 s; and finally 40°C for 30 s.

These PCRs were performed in a total volume of 25 μL, which included 10 μL sample. Reactions used uracil DNA glycosylase (Roche Diagnostics) to prevent carry over contamination. Every real-time PCR run included a positive control (*L. pneumophila* sg 1 ATCC-33152) and a nontemplate control (PCR grade water; Roche Diagnostics). Moreover, the assay included an external control (*L. pneumophila* sg 1 ATCC-33152 genomic DNA at a concentration of 100 genomic units/reaction) in a parallel reaction for every sample to monitor possible partial or total PCR inhibitions.

References

1. Benitez AJ, Winchell JM. Clinical application of a multiplex real-time PCR assay for simultaneous detection of *Legionella* species, *Legionella pneumophila*, and *Legionella pneumophila* serogroup 1. J Clin Microbiol. 2013;51:348–51. PubMed https://doi.org/10.1128/JCM.02510-12

2. Collins S, Jorgensen F, Willis C, Walker J. Real-time PCR to supplement gold-standard culture-based detection of *Legionella* in environmental samples. J Appl Microbiol. 2015;119:1158–69. PubMed https://doi.org/10.1111/jam.12911
